# Risk Factors Associated With Increased Ethically Challenging Situations Encountered by Veterinary Team Members During the COVID-19 Pandemic

**DOI:** 10.3389/fvets.2021.752388

**Published:** 2021-10-25

**Authors:** Anne Quain, Siobhan Mullan, Michael P. Ward

**Affiliations:** ^1^Sydney School of Veterinary Science, Faculty of Science, University of Sydney, Camperdown, NSW, Australia; ^2^UCD School of Veterinary Medicine, University College Dublin, Dublin, Ireland

**Keywords:** COVID-19, veterinary ethics, moral stress, veterinary nurse, animal health technician, veterinary technician, veterinarian

## Abstract

Ethically challenging situations (ECS) are commonly encountered in veterinary settings. The number of ECS encountered by some veterinary team members may increase during a crisis, such as the COVID-19 pandemic. This study aimed to determine the risk factors for experiencing an increase in the frequency of ECS in the months following the beginning of the COVID-19 pandemic, utilizing data from a global survey of veterinarians, veterinary nurses and animal health technicians collected from May to July 2020. In this study, descriptive analyses were performed to characterize veterinary team members who responded to the survey (*n* = 540). Binomial logistic regression analyses were performed to determine factors associated with an increase in ECS encountered since the beginning of the COVID-19 pandemic. Being a veterinary nurse or animal health technician, working with companion animals, working in the USA or Canada, and being not confident or underconfident in dealing with ECS in the workplace were factors associated with an increase in ECS encountered since the beginning of the COVID-19 pandemic. Results suggest a need to explore the ECS encountered by veterinary team members, particularly veterinary nurses and animal health technicians working in companion animal practice, in depth. Identification of risk factors may facilitate better preparation of veterinary team members for managing ECS, and minimizing the negative impact of ECS on the well-being of those who care for animals.

## Introduction

Ethically challenging situations (ECS) are commonly encountered by veterinary team members, and can lead to moral stress and moral distress (1–6).

During the COVID-19 pandemic, declared by the World Health Organization on 11 March 2020 (7), veterinarians, animal health technicians, veterinary nurses, and other health professionals (“veterinary team members”) encountered ECS not documented in previous surveys. A global survey of 540 veterinary team members, found that such ECS included conflict between personal well-being and professional role, and deciding what constitutes an essential veterinary service (8). They also encountered well-documented ECS, such as dealing with clients with financial limitations, and conflicts between the interests of the animal and those of the client. Of those experiencing an increase in ECS since the beginning of the COVID-19 pandemic, the median frequency of ECS encountered by respondents increased from several times per month to several times per week (Spearman rank correlation 0.619, *p* < 0.0001) (8).

Veterinary professionals, like health care professionals, must provide an appropriate standard of care to their patients, despite the risk of transmission of SARS-CoV-2 from clients and colleagues to both themselves, and potentially their household members during the global pandemic. They must therefore balance the needs of themselves and their loved ones with those of their patients and clients (9). During the early months of the pandemic in particular, risk management was complicated by uncertainty around the nature and transmissibility of SARS-CoV-2, as well as uncertainty around what veterinary services were “essential,” and which could be delayed (8, 10).

While client financial constraints represent a common ethical challenge to veterinary team members (1, 3, 6, 11, 12), the frequency and extent of financial limitations were likely exacerbated due to the economic consequences of COVID-19. These include widespread business closures, trade and supply chain disruption, absenteeism due to sickness, reduced productivity, altered consumer spending habits, and COVID-19 associated deaths (13). Financial constraints may also exacerbate conflicts between the interests of clients and the interests of their animals, another well-documented ethical challenge encountered by veterinary team members (2, 14).

Moral stress associated with ethical challenges may negatively impact the well-being of veterinary team members, contributing to overall mental health morbidity and even mortality (15–17).

Determining risk factors associated with an increased frequency of ECS encountered may be helpful in targeting interventions to better prepare veterinary team members for dealing with ECS in a crisis situation.

The objective of this study was to determine the risk factors for an increase in ECS encountered after the beginning of the COVID-19 pandemic.

## Materials and Methods

### Study Design

This study was conducted as part of a larger research project examining ECS encountered by veterinary team members since the beginning of the COVID-19 pandemic. Detailed information regarding the study design has been published elsewhere (8).

Briefly, the study entailed a survey administered to veterinarians, animal health technicians and veterinary nurses (veterinary team members) globally over a 2 month period (13 May to 14 July 2020). Veterinary team members were recruited primarily *via* social media, newsletters of veterinary organizations, industry contacts and word of mouth. Those wishing to participate could access the survey through an “open” link which could be shared with others. Participation was voluntary. No incentives were offered. Participants were provided with a Participant Information Statement and were only able to submit a response if they consented to participate. To meet the inclusion criteria, respondents were required to be a veterinarian, animal health technician, or veterinary nurse over the age of 18 years. The study was approved by the University of Sydney Human Research Ethics Committee (project 2020/291).

The mixed methods survey consisted of 29 questions across three sections. In the first section, participants were asked how frequently they experienced ECS prior to the advent of the COVID-19 pandemic. They were asked to describe the most common and the most stressful ECS that they had encountered since then. Additionally, they were asked to rate the frequency they encountered a list of different ECS. In the second section, participants were asked specific questions about the most recent ECS they had encountered. In the third section, participants were asked nine demographic questions, including their professional role, country of work, year of graduation, year of birth, gender, caseload, hours worked per week in their current role, whether they were taught ethics as part of the training toward their qualification, and whether they had undertaken any ethics training after gaining their qualification. They were also asked to rate their confidence in dealing with ECS in their workplace, and their autonomy in making and acting on ethical decisions in their workplace. The questionnaire has been published previously (8).

The survey platform utilized was Research Electronic Data Capture (REDCap), a secure web application designed for building and managing surveys, as well as data storage and export, hosted by the University of Sydney.

The current study utilized quantitative data from the first and second questions in the first section of the survey, and demographic data from the third section of the survey, to determine risk factors for experiencing an increase in the frequency of ECS with the beginning of the pandemic.

### Data Cleaning

Survey data from REDCap were downloaded into Microsoft® Excel® for Microsoft 365 MSO (16.0.13328.20262). Where respondents had selected “other” from the drop-down menu and subsequently specified a response already represented by an option in the drop-down menu, the response was recategorized as such. Only responses not reflected in the drop-down menu were retained in the “other” category. Data were checked for logical values.

The spreadsheet was imported into IBM® SPSS® Statistics Version 26 (release 26.0.0.0).

### Outcome and Explanatory Variables

The difference between the frequency of ECS encountered following the advent of the COVID-19 pandemic and prior to the pandemic was calculated, and recoded into the new binary variable “increase vs. no increase.” The variable “age” was calculated by subtracting the year of birth from 2020. The variable “experience” was calculated by subtracting the year of graduation or qualification from 2020.

A total of 11 explanatory variables were considered for regression analyses: role, gender, age, years of experience, region, hours worked, caseload, ethics training for qualification, post-qualification ethics training, confidence in resolving ECS, and autonomy in resolving ECS. All explanatory variables were used as categorical (nominal or ordinal) variables, except for two continuous variables: age and years of experience.

To facilitate statistical analysis, some variables were recoded into new variables (see Supplementary Table 1).

### Descriptive Analyses

Descriptive analyses were performed by assessing the distribution of categorical variables with frequency tables. Continuous variables were described using summary statistics and boxplots.

Contingency tables were used to describe the association between categorical variables and the binary outcome variable “increase vs. no increase.” The distribution of continuous variables by each category of the outcome variable was described with summary statistics [median, interquartile range (IQR)].

### Univariable Analyses

Univariable binary logistic regression analyses were performed to assess the association between the explanatory variables and the outcome variable. The continuous variables “age” and “years of experience” were tested for collinearity, and the assumption of linearity of log odds was assessed graphically by categorizing these variables (quartile values) and plotting the log odds.

Variables were checked for missing values. In both cases of variables with missing values (gender, *n* = 4 and age, *n* = 12), <10% of values were missing so these variables were retained for inclusion in the analysis.

### Multivariable Analyses

A forward selection approach was used to build the multivariable model. All variables with a *p*-value of <0.25 on univariable analysis were eligible for inclusion in the multivariable model. Interaction terms between variables were not considered. The best model was identified based on likelihood ratio tests and was evaluated using a Hosmer-Lemeshow goodness-of-fit statistic and Nagelkerke *R*^2^ statistic. Outliers were identified based on residual values (>2 standard deviations). Variables included in the model selected were interpreted using estimated odds ratios and 95% confidence intervals.

All statistical analyses were conducted using IBM SPSS Statistics® Version 26 (release 26, IBM Corporation).

## Results

In total, 551 veterinary team members submitted a response to the survey, of which two were test responses and nine did not contain answers to individual questions. Therefore, a total of 540 responses were analyzed.

The distribution of categorical demographic variables is described in Table 1, and continuous variables are described in Table 2. Briefly, the majority of respondents were female (*n* = 434, 80.4%) veterinarians (*n* = 423, 78.3%) working in companion animal practice (*n* = 367, 68.0%). The age of respondents ranged from 20 to 94 years, with a median of 40 (IQR 18). The years since qualification or graduation ranged from 0 to 62 years, with a median of 13 (IQR 17).

**Table 1:** Frequency table for the re-categorized demographic information on respondents to a mixed methods survey on ethically challenging situations encountered by veterinary team members globally in the early stages of the COVID-19 pandemic (*n* = 540).

**Variable**	**Category**	**Number**	**Percentage %**
Gender	Female	434	80.4
	Male	102	18.9
	Other	4	0.7
Role	Veterinarian	423	78.3
	Other	117	21.7
Hours worked	0–30	116	21.5
	31–40	186	34.4
	41–>50	238	44.1
Caseload	Companion animal clinical practice	367	68.0
	Other clinical practice	103	19.0
	Non-clinical role	70	13.0
Ethics education undertaken as part of qualification/degree	Yes	293	54.3
	No	161	29.8
	Don't recall	86	15.9
Ethics education or training to any degree following qualification/degree	Yes	280	51.9
	No	260	48.1
Region/group of countries	Australia and New Zealand	328	60.7
	USA and Canada	151	28.0
	Other: EU, Asia, Caribbean, Africa	61	11.3
Confidence in resolving ECS	Not confident at all/underconfident	79	14.6
	Confident enough that I can get by	231	42.8
	Reasonably confident/couldn't be more confident	230	42.6
Autonomy	Never/rarely	50	9.3
	Sometimes	110	20.4
	Most of the time/always	380	70.4

**Table 2:** Descriptive information for continuous explanatory variables classified by the outcome variable increase vs. no increase in ethically challenging situations encountered by veterinary team members in the early months of the COVID-19 pandemic (*n* = 540).

	**ECS**	**Minimum**	**25th percentile**	**Median**	**75th percentile**	**Maximum**	***p*-value**
Age							0.001
	Increase	20	30.00	37.00	48.00	94	
	No change	22	33.00	41.00	50.00	86	
	Total	20	31.00	40.00	49.00	94	
Experience							<0.001
	Increase	0	5.00	10.00	20.75	50	
	No change	0	7.00	15.00	24.00	62	
	Total	0	6.00	13.00	23.00	62	

Just under half worked more than 41 h per week (*n* = 238, 44.1%) while around one third worked 31–40 h per week (*n* = 186, 34.4%). More than half of the respondents were based in Australia or New Zealand (*n* = 328, 60.7%). Just over half (*n* = 293, 54.3%) had some form of ethics education as part of their qualification or degree, and slightly fewer (*n* = 280, 51.9%) had undertaken some form of ethics education after qualifying or graduating. The majority were confident that they could resolve ECS, with 42.8% (*n* = 231) reporting that they were confident enough to get by, while 42.6% (*n* = 230) reported they were either reasonably confident or couldn't be more confident in dealing with ECS in their workplace. The majority (*n* = 380, 70.4%) reported that they were free to make and act on ethical decisions in their workplace most of the time or always.

### Factors Associated With Increased Ethically Challenging Situations Encountered Since the Advent of the COVID-19 Pandemic

Of the participating veterinary team members, almost half (*n* = 256, 47.4%) encountered an increase in ECS since the beginning of the COVID-19 pandemic.

Eleven variables were included in the univariable analysis, of which nine were associated (*p* <0.25) with an increase in ECS encountered: age, experience, role, gender, region, hours worked, caseload, confidence in resolving ECS, and autonomy in decision making (Tables 2, 3). No variables were excluded due to missing values. The variables “age” and “experience” were highly correlated (*r* = 0.93). “Experience” was included in the final model because it was more strongly associated with the outcome (*p* < 0.001). As age increased, participants were less likely to experience an increase in ECS. In the univariable model, for each 1 year increase in age, the odds of an increased ECS decreased by 2.4% (95% CI: 0.1–3.9%). As experience increased, participants were less likely to experience an increase in ECS. For each 1 year increase in experience, the odds of an increased ECS decreased by 2.7% (95% CI: 1.2–4.2).

**Table 3:** Contingency tables and univariable logistic regression results for demographic variables associated with an increase in ethically challenging situations encountered since the beginning of the COVID-19 pandemic, in a global survey of veterinary team members (*n* = 540).

**Variable category**	**Increased ECS**		***B***	**SE(*b*)**	**Odds ratio**	**95% CI**	***p*-value**
	**Increased (row %)**	**No change or decreased (row %)**					
**Role**						
Veterinarian	181 (42.8)	242 (57.2)	−0.87	0.22	0.4	0.3; 0.6	<0.001
Nurse/Technician/other	75 (64.1)	42 (35.9)	0	–	1	–	–
**Gender**						
Female	216 (49.8)	218 (50.2)	0.55	0.23	1.7	1.1;2.8	0.015
Male	37 (36.3)	65 (63.7)	0	–	1	–	–
Missing: 4							
**Region/group of countries**							0.001
Australia and NZ	135 (41.2)	193 (58.8)	0	–	1	–	–
USA and Canada	89 (58.9)	62 (41.1)	0.72	0.20	2.1	1.4;3.0	–
Other: EU, Asia, Caribbean, Africa	32 (52.5)	29 (47.5)	−0.46	0.28	1.6	0.9; 2.7	–
**Hours worked**							0.088
0–30 h/week	46 (39.7)	70 (60.3)	−0.30	0.23	0.7	0.5;1.2	–
31–40 h/week	98 (52.7)	88 (47.3)	0.23	0.20	1.3	0.9; 1.8	–
41–50 h/week	112 (47.1)	126 (52.9)	0	–	1	–	–
**Caseload**							<0.001
Companion animal practice	197 (53.7)	170 (46.3)	1.06	0.29	2.9	1.7;5.0	-
Clinical practice (non-companion animal)	39 (37.9)	64 (62.1)	0.42	0.33	1.5	0.8; 2.9	-
Other	20 (28.6)	50 (71.4)	0		1		–
**Ethics training for qualification**							0.959
Yes	138 (47.1)	155 (52.9)	−0.07	0.25	0.9	0.6; 1.5	–
No	76 (47.2)	85 (52.8)	−0.66	0.27	0.9	0.6;1.6	–
Don't recall	42 (48.8)	44 (51.2)	0	–	1	–	–
**Post-qualification ethics training**							0.519
Yes	127 (48.8)	133 (51.2)	0.11	0.17	1.1	0.8; 1.6	–
No	129 (46.1)	151 (53.9)	0	–	1	–	–
**Confidence in resolving ECS**							0.001
Not confident at all/underconfident	48 (60.8%)	31 (39.2%)	0.92	0.27	2.5	1.5; 4.2	–
Confident enough that I can get by	120 (51.9%)	111 (48.1%)	0.56	0.19	1.7	1.2; 2.5	–
Reasonably confident/couldn't be more confident	88 (38.3%)	142 (61.7%)	0	–	1	–	–
**Autonomy in decision making**							0.001
Never/Rarely	33 (66.0)	17 (34.0)	0	–	1	–	–
Sometimes	62 (56.4)	48 (43.6)	−0.41	0.36	0.7	0.3; 1.3	–
Mostly/always	161 (42.4)	219 (57.6)	−0.97	0.32	0.4	0.2; 0.7	–

The final multivariable logistic regression model for the increase in ECS in veterinary team members is presented in Table 4. Respondents who were not veterinarians (OR 2.2, 95% CI 1.4–3.4), those who worked in companion animal practice (OR 3.2, 95% CI 1.7–5.8), those working in the USA or Canada (OR 2.4, 95% CI 1.6–3.7) and those who were not confident at all or underconfident in resolving ECS (OR 2.4, 95% CI 1.4–4.2) were more likely to experience an increase in the frequency of ECS at the beginning of a global pandemic, compared to respondents who were veterinarians, who worked in non-clinical practice, who worked in Australia and New Zealand and who were reasonably confident or couldn't be more confident in managing ECS, respectively. Hosmer-Lemeshow goodness-of-fit Chi-squared *p*-value (*p* = 0.310) indicated adequate model fit. Nagelkerke *R*^2^ was 0.151. Examination of model residuals (using Studentized residuals >2.0) did not reveal any systemic lack of model fit. No confounding by age or gender was found in the final model selected.

**Table 4:** Final binary multivariable logistic regression model of risk factors for an increase in ethically challenging situations encountered, in a global survey of veterinary team members at the advent of the COVID-19 pandemic (May-July 2020) (*n* = 540).

**Variable category**	***B***	**SE(*b*)**	**Adjusted odds ratios**	**95% CI**	***p*-value**
**Role**					<0.001
Veterinarian	0.00	–	1.0	–	–
Non-veterinarian	0.78	0.23	2.2	1.4–3.4	–
**Caseload**					<0.001
Non-clinical	0.00	–	1.0	–	–
Companion animal practice	1.15	0.31	3.2	1.7–5.8	–
Other clinical practice	0.58	0.36	1.8	0.9–3.5	–
**Region/group of countries**					<0.001
Australia and New Zealand	0.00	–	1.0	–	–
USA and Canada	0.88	0.22	2.4	1.6–3.7	–
Other: EU, Asia, Caribbean, Africa	0.61	0.30	1.8	1.0–3.3	–
**Confidence**					0.001
Not confident/underconfident	0.88	0.28	2.4	1.4–4.2	–
Confident enough that I can get by	0.58	0.20	1.8	1.2–2.6	–
Reasonably confident/couldn't be more confident	0.00	–	1.0	–	–

## Discussion

This study explored factors associated with a reported increase in ethically challenging situations encountered by veterinary team members during the early months of the COVID-19 pandemic (May to July) in 2020. Demographic factors associated with a higher frequency of ECS were being a veterinary nurse or animal health technician; working in companion animal practice; working in the USA or Canada; and degree of confidence in dealing with ECS.

### Being a Veterinary Nurse or Animal Health Technician

Veterinary nurses and animal health technicians were 2.2 times more likely to experience an increase in ECS than veterinarians early in the COVID-19 pandemic. Most surveys on ECS in the veterinary literature focus on the experiences of veterinarians (1, 2, 5, 6). Lehnus et al. included anesthesia nurses or technicians in their survey of veterinary anesthetists (6.0%), but did not differentiate responses based on professional role (4). Moses et al. found that cases where a veterinarian felt they could not do the “right thing” caused some degree of distress to 97.7% of staff, and “inappropriate” requests for euthanasia caused some degree of distress in 96.1% of staff, where staff may have included veterinarians, animal health technicians, veterinary nurses and other veterinary team members (3). In the same study, while 64.7% of respondents never or rarely had disagreements with non-veterinarian staff about how to proceed with a clinical case, 32.3% sometimes disagreed, and 2.9% often or always disagreed. These findings suggest that ECS are a concern for all veterinary team members, not just veterinarians. A survey of equine veterinarians, veterinary nurses, and veterinary students undertaken in June 2020 in the UK (*n* = 451) reported lower levels of mental well-being among veterinary nurses than veterinarians (18). The authors do not speculate on the reasons for this difference, but suggest that they point to a need for support strategies to target this cohort. Our findings confirm and strengthen the need for strategies to support veterinary nurses, animal health technicians and non-veterinarian team members in managing ECS.

In a study of Canadian veterinarians (*n* = 537) and animal health technicians (*n* = 453), autonomy was effective in reducing co-worker strain, but was less common in female animal health technicians, the lowest status team members (19). We speculated that low autonomy would be associated with an increase in ECS encountered, however this was not supported in our final multivariable model.

A study of veterinary technicians (*n* = 256) across four veterinary teaching hospitals in the USA and Canada undertaken prior to the pandemic found higher rates of burnout than in a comparable group of trauma nurses (20). Burnout was associated with feelings of fear or anxiety regarding supervisor communications, a perception that the caseload was too high to permit excellent patient care, and a perception of lack of assistance during sudden workload increases, all of which may lead to moral distress. These conditions were also present during the pandemic. For example, the overall caseload for emergency clinics in the USA increased by >10%, while 44% of hospitals reported caseload increases of >25% (21). Despite these increases, the majority of hospitals did not increase staff levels, and many suffered staff shortages due to potentially COVID-19 exposed staff isolating, sickness absenteeism and other COVID-19 related absences including childcare, home-schooling and being unwilling to work (21, 22). With the majority of practices changing operations to minimize contact with clients (23), it is likely that many veterinary nurses and animal health technicians had more interaction with clients than veterinarians, which may account for an increase in ECS. It is also possible that, due to staff shortages, veterinary nurses and animal health technicians found themselves performing duties they may not have previously performed, such as ensuring clients followed biosecurity protocols, triaging patients, and undertaking extensive deep cleaning of the workplace environment. These issues were reflected in the free text responses to our survey questions (8).

While a lack of ethics training was not significantly associated with an increase in ECS encountered, this study could not differentiate the impact of the quality or quantity of ethics training. It is still possible that targeted training may assist veterinary nurses and animal health technicians in navigating ECS.

### Working in Companion Animal Practice

Veterinary team members working in companion animal practice were 3.2 times more likely to experience ECS than those working in non-clinical roles, and 1.4 times more likely to experience ECS than those in other clinical practice (for example large animal, mixed, or zoological). This may be because the onset of the pandemic was followed by an increase in companion animal adoptions (24, 25). Because people increasingly share their home with companion animals, they may have been more attentive to health problems when following stay at home orders. These factors, combined with staff shortages, may have contributed to the increased caseloads of facilities providing care to companion animals (21, 22). We did not ask respondents to specify their location, nor whether they worked in a metropolitan or regional area. It is possible that companion animal practices were more likely to be located in metropolitan areas, where social distancing was more difficult.

Ethical challenges may be encountered more commonly in companion animal practice, as companion animals are increasingly treated like family members, yet in most jurisdictions remain the legal property of the owner. They are both moral subjects and objects, and therefore occupy a unique place in veterinary ethics (26). Unlike livestock and laboratory animal practice, for example, companion animal practice is “patient-centered,” with a focus on the “best interests of the patient” rather than the benefit of the users or consumers of animals (27). Thus, there is more potential for conflict between the interests of the patient and the interests of the client. Furthermore, due to increased specialization and the availability of advanced veterinary care, costs of companion animal care have increased at a greater rate than those of production animals, where operations are increasingly streamlined to reduce costs (28). These trends generate ethical challenges, including whether to perform an advanced and potentially costly procedure (27). In addition, companion animal euthanasia has been documented as a source of moral distress among veterinary team members (2, 3, 16). It is possible that economic consequences of the pandemic (for example, increased unemployment) may have contributed to increased rates of “economic euthanasia” (29). Concerns about a perceived increase in economic euthanasia were raised by a number of respondents [see (8), Supplementary Material].

While pet insurance may protect against economic euthanasia (30, 31), many veterinary patients are uninsured. It is possible that in times of economic hardship, clients who have pet insurance may not be able to afford to pay for continued cover. There may be scope for pet insurance providers to enable policy holders to temporarily reduce their cover, or to defer payments for a limited period due to economic hardship, but such measures must be sustainable. Third-party credit is often contingent on employment status, making this option unavailable to clients unemployed due to COVID-19, or indeed other factors. Veterinary practices may not have been able to offer clients credit due to their own cash shortfalls. In April 2020 in the USA, more than 60% of practices applied for Small Business Administration loan programs, nearly 60% of practice owners forewent their own salaries, and around 60% withdrew from cash reserves (23). In such circumstances, the availability of low-interest, long-term loans to companion animal owners may help reduce economic euthanasia. Such a scheme could be funded *via* donations or a small levy paid by pet owners with the means to do so, and administered by veterinary professional organizations.

In times of widespread economic disruption and hardship, it is important for veterinary teams to be able to offer clients options along a spectrum of care (32). To this end, it is important that veterinary teams are trained and equipped to offer a spectrum of veterinary care (33).

### Working in the USA or Canada

Early in the pandemic, veterinary team members in the regions or groups of countries “USA and Canada,” and “Other: EU, Asia, Caribbean, Africa,” were 2.4 and 1.8 times more likely, respectively, to encounter an increase in ECS compared to those in “Australia and New Zealand.”

Differences in the intensity of the impact of COVID-19 between regions or groups of countries may be due to different case numbers as well as the timing and nature of policy responses, the duration and severity of lockdown and mobility restrictions and economic factors, including social security (34). In the period since the pandemic began until 31 July 2020, the USA recorded 4,388,566 cases with 150,054 deaths, and Canada recorded 115,470 cases with 8,917 deaths (35). During the same period, Australia reported only 16,905 cases with 196 deaths (36), and New Zealand 1,518 cases and six deaths (37).

However, factors specific to different regions or groups of countries may contribute to differences in risk of an increase in the frequency of ECS between different regions or groups of countries. For example, between 26 May—the day immediately following George Floyd's death—and 22 August, there were more than 7,750 demonstrations linked to the Black Lives Matter movement across over 2,440 locations in the USA alone (38). These demonstrations were associated with widespread social disruption. While it is possible that these had impacts on veterinary team members living and working in areas the demonstrations took place, the extent of the impact on frequency and type of ECS encountered by veterinary team members is not known.

It is possible that during the survey period (May to July 2020), practices in North America were busier than in other regions or groups of countries, which may have impacted the frequency of ECS encountered. An online survey of 4,105 dog owners found that while 22.3% reported that their dog had needed veterinary care in the early months of the COVID-19 pandemic, and 79.8% of those had been presented for veterinary care, the percentage was higher in some countries (for example, 24% in the US) compared with others (for example, 13% in the UK) (10). An online survey of 956 cat owners found that 17% reported that their cat needed veterinary care in the early months of the COVID-19 pandemic, and 70.9% presented their cat to a veterinarian (39). The main reasons for seeking veterinary attention at this time were monitoring an illness or disease (26.7%), wellness exams (22.3%), and vaccinations (19.6%). According to surveys performed by the AVMA in April and July 2020, while practices experienced a decrease in client traffic in April, by July, almost half of practices surveyed saw an increase of 10–30% client traffic when compared to the previous year (23). In contrast, data from the UK's Small Animal Veterinary Surveillance Network (SAVSNET) based on electronic health records from 500 veterinary sites and 10 veterinary diagnostic laboratories in the United Kingdom (representing 15 and 50% of available data, respectively) initially recorded an 80–90% reduction in use of veterinary services compared to the same time in 2019 (40). The usage of veterinary services increased subsequently, but remained at around 45–50% by July (41–43).

### Level of Confidence

Respondents who reported that they were not confident at all or underconfident in managing ECS were 2.4 times as likely to encounter an increase in ECS than those who were reasonably confident or couldn't be more confident. This suggests that increasing confidence in managing ECS would benefit veterinary team members. Again, while prior ethics training was not significantly associated with an increase in ECS in this study, we did not evaluate the quality or quantity of ethics training. A small study comparing the moral reasoning of qualified veterinarians (*n* = 65) with members of the public (*n* = 33) in the UK identified a large variation in the moral reasoning of veterinarians (44). Practicing veterinarians (*n* = 38) had moral reasoning abilities that were no better than those of the general public, and did not improve with years of experience, suggesting that veterinary training itself may not be sufficient in guiding veterinarians to manage ECS. In a survey of veterinarians in the USA (*n* = 484), 51% had received ethics training (11). Of these, only 39% agreed that this prepared them to manage ECS, 38% were neutral and 23% disagreed. Respondents to the current study had similar rates of ethics training, with 54.3% of respondents undertaking ethics training as part of their qualification, and 51.9% undertaking some form of ethics education following their qualification, for example, as part of continuing professional development. Improving the quality and quantity of ethics training available to veterinary team members, both pre and post-qualification or certification, may help veterinary team members better manage ECS and associated moral stress.

The most common resource utilized by veterinary team members facing ECS was discussion with colleagues (*n* = 341, 63.1%), followed by workplace policies (*n* = 174, 32.2%) (8). A qualitative study of Australian small animal veterinarians revealed that veterinarians valued and relied on their peers for ethical discussions and support in the face of ethical challenges (45). However, some participants feared being negatively judged by their peers, and as such colleagues could act as both a source of support as well as a source of stress or anxiety for veterinary team members. Discussions about ethically challenging situations require a high degree of trust, and a facilitator who is both knowledgeable and sensitive (45). Structured ethical debriefing, or “ethics rounds,” has the potential to increase confidence in managing ECS by improving ethical awareness, moral reasoning skills, ethical climate, and communication around what can be contentious issues in a psychologically safe space (46–48). It is possible that ECS disclose systemic issues that need to be addressed. For example, veterinary team members repeatedly faced with ECS regarding how to proceed when clients have financial limitations may benefit from clear workplace policies (49). Similarly, ECS such as conflict between personal well-being and professional role and whether to perform non-contact consultations may be reduced by clear workplaces policies and guidelines regarding biosecurity, together with team and client education and consistent messaging.

While it seems intuitive that those who are underconfident may encounter ECS more frequently, it is also possible that for some respondents, that a low confidence rating may reflect recall bias secondary to a negative encounter with an ECS.

### Factors That Were Not Associated With an Increased Risk of Encountering ECS

Our multivariable logistic regression model did not support gender, hours worked, experience, or autonomy as risk factors for encountering increased ECS since the beginning of the global pandemic. Previous studies suggest a complex relationship between gender and ECS. While a significant gender difference was detected in stress ratings of two ethical challenges, with female veterinarians in the UK rating these more stressful than their male counterparts, there was no effect of gender on the number of ECS reported (44). Similarly, while gender did not predict reports of more frequent ECS, female veterinarians in the USA were over three times as likely as their male counterparts to consider ECS a leading source of stress in their work (2). These trends require exploration with further qualitative studies.

We anticipated that an increase in hours worked would be correlated with an increase in the frequency of ECS encountered by veterinary team members at the onset of the pandemic, because veterinary team members working longer hours may be exposed to more ECS. This was not supported in our final model. However, this study did not capture changes in working hours associated with the pandemic. Many veterinary services reduced operating hours, for example in the USA and UK (21–23, 40–43, 50), and a reduction in working hours may have reduced the risk of an increase in ECS encountered.

The role of experience in the frequency and stressfulness of ECS encountered by veterinary team members remains unclear. Our findings align with a study of UK veterinarians, which found was no statistically significant relationship between years in practice and stress associated with ECS (6). In contrast, a study of veterinarians in the USA, veterinarians with under 15 years experience were almost 2.5 times more likely to report frequent ethical dilemmas than their more experienced counterparts (2). While a UK study found that moral reasoning among veterinarians did not improve with experience (44), further studies are required to determine whether this precludes improved recognition and management of ECS. In addition, further studies are required to determine whether experience mitigates the risk with regards to some ECS, and not others.

As discussed, we anticipated that low autonomy would be associated with an increase in the frequency of ECS encountered by veterinary team members, based on previous reports of low autonomy associated with occupational stress in veterinary settings (19). In addition, the likelihood of reporting frequent ECS was over 1.8 times greater in associates, a lower autonomy position, than practice owners (2). However, it is possible that low autonomy was associated with an increase in stress associated with ECS, however we did not examine this outcome.

## Conclusion

Being a veterinary nurse or animal health technician, working with companion animals, working in the USA or Canada, and being not confident or underconfident in dealing with ECS in the workplace were factors associated with experiencing an increase in ECS encountered since the beginning of the COVID-19 pandemic.

Further studies are required to assess the impact of interventions such as ethics debriefing, policies and guidelines on the ability of veterinary team members to manage ECS.

## Strengths and Limitations

This study is the largest global survey of ECS encountered by veterinary teams to date, and the first global survey to document ECS encountered by veterinary teams during a pandemic. It was conducted during the initial months of the COVID-19 pandemic, when the majority of people from respondent's countries were subject to public health restrictions impacting all aspects of their lives.

This sample does not represent a random sample and, as an online survey, is biased toward internet and social media users willing to complete surveys. The survey link was seen by an unknown number of individuals, precluding denominator data to calculate a response rate. The non-random, convenience sampling method may have biased selection toward respondents who had strong views or experiences relating to ECS, or biased selection toward particular groups. For example, the majority of organizations who agreed to share the survey link were organizations regulating or representing veterinarians, as opposed to veterinary nurses and animal health technicians [see (8) for a complete list of these organizations]. The convenience sampling method and number of responses from the majority of countries, particularly low and middle-income countries, was too small to permit direct comparisons between countries, which could have provided valuable insights.

While we attempted to group countries according to region, those in the category “other” (EU, Asian, Caribbean, Africa) were grouped to facilitate statistical analysis, and are not necessarily in the same geographic region. Therefore, comparisons between “other” and the regions need to be interpreted with caution.

A handful of countries were overrepresented, while the majority of countries were not represented at all. The results are biased toward wealthy, Western countries, where the majority of veterinarians work with companion animals. Therefore, this study may have failed to capture the types and frequency of ECS encountered in other contexts. For example, a study of the impact of COVID-19 on the Working Equid Community found that equid owners reported decreased equid workload, decreased equid derived income and decreased household income, in the context of unchanged or increasing costs of equid related services, and in 15% of cases, reduced availability of these services (51). Any or all of these factors may have been associated with changes in the frequency of ECS encountered by veterinary team members.

Open surveys are associated with the risk that respondents may misrepresent themselves or complete the survey multiple times, or that web robots may generate responses (52). All responses were reviewed, and all included responses contained unique, detailed information suggesting that the data are legitimate.

The survey was anonymous to maximize protection of respondent's privacy. A major disadvantage of anonymity is the inability to clarify responses, or follow up. Additionally, we were unable to support individuals expressing strong negativity, other than providing very general information about support services at the conclusion of the survey (53). While our respondents were able to expand on their answers to some extent in the free-text comments, it would have been ideal to interview respondents in order to achieve a more comprehensive understanding of factors leading to an increase in ECS encountered at the beginning of the pandemic. However, by ensuring anonymity we believe that responses were frank and reflected the reality of respondent experience.

The survey was extended, and administered at a time when respondents were time-poor and potentially burnt out (21). A briefer survey may have captured a greater number and therefore breadth of responses.

Nonetheless, while drawn from a non-representative sample of participants, the wide representation of veterinary team members and representativeness among demographics including age, experience and case load indicates a meaningful range of responses.

Finally, these results provide a snapshot of ECS encountered by veterinary team members during a limited period (May to July 2020). This was a time when many countries were experiencing the first wave of the pandemic, public health measures such as social distancing, mask wearing and lockdowns were unprecedented, variants had not yet been identified, and vaccines were not yet available (54). Longitudinal studies would be required to document changes in the frequency, type and stressfulness of ECS encountered by veterinary team members through the course of an extended global pandemic.

## Data Availability Statement

The datasets presented in this article are not readily available because we have approval to disseminate aggregated data, but not individual data. Requests to access the datasets should be directed to anne.quain@sydney.edu.au.

## Ethics Statement

The studies involving human participants were reviewed and approved by the University of Sydney Human Research and Ethics Committee (approval number 2020/291). Participants provided their written informed consent to participate in this study.

## Author Contributions

AQ: literature review, study design, survey building and piloting, ethics application, data analysis, writing, and editing submission. SM: study design, survey refinement, ethics application, editing, and supervision. MW: data analysis, editing, and supervision. All authors contributed to the article and approved the submitted version.

## Conflict of Interest

The authors declare that the research was conducted in the absence of any commercial or financial relationships that could be construed as a potential conflict of interest.

## Publisher's Note

All claims expressed in this article are solely those of the authors and do not necessarily represent those of their affiliated organizations, or those of the publisher, the editors and the reviewers. Any product that may be evaluated in this article, or claim that may be made by its manufacturer, is not guaranteed or endorsed by the publisher.

## References

[1] Crane MF Phillips JK Karin E. Trait perfectionism strengthens the negative effects of moral stressors occurring in veterinary practice. Aust Vet J. (2015) 93:354–60. 10.1111/avj.12366 26412116

[2] Kipperman B Morris P Rollin B. Ethical dilemmas encountered by small animal veterinarians: characterisation, responses, consequences and beliefs regarding euthanasia. Vet Record. (2018) 182:104619. 10.1136/vr.104619 29445010

[3] Moses L Malowney MJ Wesley Boyd J. Ethical conflict and moral distress in veterinary practice: a survey of North American veterinarians. J Vet Intern Med. (2018) 32:2115–22. 10.1111/jvim.15315 30320478 PMC6271308

[4] Lehnus KS Fordyce PS Mcmillan MW. Ethical dilemmas in clinical practice: a perspective on the results of an electronic survey of veterinary anaesthetists. Vet Anaesth Analg. (2019) 46:260–75. 10.1016/j.vaa.2018.11.006 30952440

[5] Duernberger C. I Would like to, but I can't. An online survey on the moral challenges of German Farm Veterinarians. J Agri Environ Ethics. (2020) 33:447–60. 10.1007/s10806-020-09833-0

[6] Batchelor CEM Mckeegan DEF. Survey of the frequency and perceived stressfulness of ethical dilemmas encountered in UK veterinary practice. Vet Record. (2012) 170. 10.1136/vr.100262 22084032

[7] Ghebreyesus TA. WHO Director-General's Opening Remarks at the Media Briefing on COVID-19 - 11 March 2020. (2020). World Health Organisation. Available online at: https://www.who.int/dg/speeches/detail/who-director-general-s-opening-remarks-at-the-media-briefing-on-covid-19-−11-march-2020 (accessed April 7, 2020).

[8] Quain A Mullan S Mcgreevy PD Ward MP. Frequency, stressfulness and type of ethically challenging situations encountered by veterinary team members during the COVID-19 pandemic. Front Vet Sci. (2021) 8:647108. 10.3389/fvets.2021.647108 33912607 PMC8071942

[9] Donkers MA Gilissen VJHS Candel MJJM Van Dijk NM Kling H Heijnen-Panis R. Moral distress and ethical climate in intensive care medicine during COVID-19: a nationwide study. BMC Med Ethics. (2021) 22:73. 10.1186/s12910-021-00641-3 34139997 PMC8211309

[10] Kogan LR Erdman P Bussolari C Currin-Mcculloch J Packman W. The initial months of COVID-19: dog owners' veterinary-related concerns. Front Vet Sci. (2021) 8:629121. 10.3389/fvets.2021.629121 33604366 PMC7884340

[11] Kipperman BS Kass PH Rishniw M. Factors that influence small animal veterinarians' opinions and actions regarding cost of care and effects of economic limitations on patient care and outcome and professional career satisfaction and burnout. J Am Vet Med Assoc. (2017) 250:785–94. 10.2460/javma.250.7.785 28306486

[12] Dürnberger C. Am I actually a veterinarian or an economist? Understanding the moral challenges for farm veterinarians in Germany on the basis of a qualitative online survey. Res Vet Sci. (2020) 133:246–50. 10.1016/j.rvsc.2020.09.029 33035930

[13] Pak A Adegboye OA Adekunle AI Rahman KM Mcbryde ES Eisen DP. Economic consequences of the COVID-19 outbreak: the need for epidemic preparedness. Front Public Health. (2020) 8:241. 10.3389/fpubh.2020.00241 32574307 PMC7273352

[14] Tannenbaum J. Veterinary medical ethics: a focus of conflicting interests. J Soc Iss. (1993) 49:143–56. 10.1111/j.1540-4560.1993.tb00914.x

[15] Arbe Montoya AI Hazel S Matthew SM Mcarthur ML. Moral distress in veterinarians. Vet Record. (2019) 2019:105289. 10.1136/vr.105289 31427407

[16] Rollin BE. Euthanasia, moral stress, and chronic illness in veterinary medicine. Vet Clin North Am Small Anim Pract. (2011) 41:651–9. 10.1016/j.cvsm.2011.03.005 21601753

[17] Moir FM Van Den Brink ARK. Current insights in veterinarians' psychological wellbeing. N Z Vet J. (2020) 68:3–12. 10.1080/00480169.2019.1669504 31607215

[18] Mair TS Mountford DR Radley R Lockett E Parkin TD. Mental wellbeing of equine veterinary surgeons, veterinary nurses and veterinary students during the COVID-19 pandemic. Equine Vet Educ. (2021) 33:15–23. 10.1111/eve.13399

[19] Wallace J Buchanan T. Status differences in interpersonal strain and job resources at work: a mixed methods study of animal health-care providers. Int J Confl Manag. (2019) 31:287–308. 10.1108/IJCMA-08-2019-0135

[20] Hayes GM Lalonde-Paul DF Perret JL Steele A Mcconkey M Lane WG. Investigation of burnout syndrome and job-related risk factors in veterinary technicians in specialty teaching hospitals: a multicenter cross-sectional study. J Vet Emerg Crit Care. (2019) 2019:12916. 10.1111/vec.12916 31840933 PMC7003767

[21] Wayne A Rozanski E. The evolving response by emergency veterinary hospitals during the COVID-19 pandemic. J Vet Emerg Crit Care. (2020) 30:601. 10.1111/vec.12995 32864824

[22] Wayne AS Rozanski EA. Cataloguing the response by emergency veterinary hospitals during the COVID-19 pandemic *via* weekly surveys. J Vet Emerg Crit Care. (2020) 30:493–7. 10.1111/vec.12974 32598096 PMC7361864

[23] AVMA. COVID-19 Impact On Veterinary Practices. American Veterinary Medical Association. (2020). Available online at: https://www.avma.org/resources-tools/animal-health-and-welfare/covid-19/covid-19-impact-veterinary-practices (accessed June 9, 2021).

[24] Morgan L Protopopova A Birkler RID Itin-Shwartz B Sutton GA Gamliel A. Human–dog relationships during the COVID-19 pandemic: booming dog adoption during social isolation. Human Soc Sci Commun. (2020) 7:155. 10.1057/s41599-020-00649-x

[25] Baptista J Blache D Cox-Witton K Craddock N Dalziel T De Graaff N. Impact of the COVID-19 pandemic on the welfare of animals in Australia. Front Vet Sci. (2021) 7:621843. 10.3389/fvets.2020.621843 33585609 PMC7876268

[26] Yeates J Savulescu J. Companion animal ethics: a special area of moral theory and practice? Ethical Theor Moral Pract. (2017) 20:347–59. 10.1007/s10677-016-9778-6

[27] Grimm H Bergadano A Musk GC Otto K Taylor PM Duncan JC. Drawing the line in clinical treatment of companion animals: recommendations from an ethics working party. Vet Record. (2018) 2018:104559. 10.1136/vr.104559 29602799 PMC6035488

[28] Gyles C. Advances in veterinary practice. Can Vet J. (2016) 57:811–2. 27493278 PMC4944555

[29] Kipperman B. Economic Euthanasia: A Disease In Need of Prevention. HSVMA. (2010). Available online at: https://www.hsvma.org/economic_euthanasia_disease_in_need_of_prevention#.W8unf2gzZPY (accessed October 21, 2018).

[30] Boller M Nemanic TS Anthonisz JD Awad M Selinger J Boller EM. The effect of pet insurance on presurgical euthanasia of dogs with gastric dilatation-volvulus: a novel approach to quantifying economic euthanasia in veterinary emergency medicine. Front Vet Sci. (2020) 7:590615. 10.3389/fvets.2020.590615 33364255 PMC7752994

[31] Anderson S Stevenson MA Boller M. Pet health insurance reduces the likelihood of pre-surgical euthanasia of dogs with gastric dilatation-volvulus in the emergency room of an Australian referral hospital. N Z Vet J. (2021) 69:267–73. 10.1080/00480169.2021.1920512 33896404

[32] Block G. A new look at standard of care. J Am Vet Med Assoc. (2018) 252:1343–4. 10.2460/javma.252.11.1343 29772971

[33] Fingland RB Stone LR Read EK Moore RM. Preparing veterinary students for excellence in general practice: building confidence and competence by focusing on spectrum of care. J Am Vet Med Assoc. (2021) 259:463–70. 10.2460/javma.259.5.463 34388008

[34] Bailey D Clark J Colombelli A Corradini C De Propris L Derudder B. Regions in a time of pandemic. Reg Stud. (2020) 54:1163–74. 10.1080/00343404.2020.1798611

[35] World Health Organisation. Coronavirus Disease (COVID-19) Situation Report – 193. World Health Organisation. (2020). Available online at: https://www.who.int/docs/default-source/coronaviruse/situation-reports/20200731-covid-19-sitrep-193.pdf?sfvrsn=42a0221d_4 (accessed July 13, 2021).

[36] Australian Government Department of Health. Coronavirus (COVID-19) at a Glance 31 July 2020. (2020). Available online at: https://www.health.gov.au/resources/publications/coronavirus-covid-19-at-a-glance-31-july-2020 (accessed July 13, 2021).

[37] New Zealand Government. Covid Cases in New Zealand (Cumulative). Stats NZ. (2021). Available online at: https://www.stats.govt.nz/experimental/covid-19-data-portal (accessed July 13, 2021].

[38] The Armed Conflict Location and Event Data Project. Demonstrations and political violence in America: New Data for Summer 2020. ACLED. (2020). Available online at: https://acleddata.com/#/dashboard (accessed July 13, 2021).

[39] Kogan LR Erdman P Currin-Mcculloch J Bussolari C Packman W. The impact of COVID on cat guardians: veterinary issues. Animals. (2021) 11:30603. 10.3390/ani11030603 33668841 PMC7996145

[40] SAVSNET. Impact of COVID-19 on Companion Animal Veterinary Practice: Report 1. 1 ed. Small Animal Veterinary Surveillance Network: University of Liverpool (2020).

[41] SAVSNET. Impact of COVID-19 on Companion Animal Veterinary Practice: Report 2. 2 ed. Small Animal Surviellance Network: University of Liverpool (2020).

[42] SAVSNET. Impact of COVID-19 on Companion Animal Veterinary Practice: Report 3. 3 ed. Small Animal Surviellance Network: University of Liverpool (2020).

[43] SAVSNET. Impact of COVID-19 on Companion Animal Veterinary Practice: Report 4. 4 ed. Small Animal Veterinary Surveillance Network: University of Liverpool (2020).

[44] Batchelor CEM Creed A Mckeegan DEF. A preliminary investigation into the moral reasoning abilities of UK veterinarians. Vet Record. (2015) 177:102775. 10.1136/vr.102775 26109285

[45] Richards L Coghlan S Delany C. “i had no idea that other people in the world thought differently to me”: ethical challenges in small animal veterinary practice and implications for ethics support and education. J Vet Med Educ. (2020) 13:e20190013. 10.3138/jvme.2019-0013 32053051

[46] Silén M Ramklint M Hansson MG Haglund K. Ethics rounds: an appreciated form of ethics support. Nurs Ethics. (2014) 23:203–13. 10.1177/0969733014560930 25527354

[47] Schmitz D Gross D Frierson C Schubert GA Schulze-Steinen H Kersten A. Ethics rounds: affecting ethics quality at all organisational levels. J Med Ethics. (2018) 44:805–9. 10.1136/medethics-2018-104831 30154217

[48] Watts L Parker L Scicluna H. Rural ethics ward rounds: enhancing medical students' ethical awareness in rural medicine. Austr J Rural Health. (2013) 21:128–9. 10.1111/ajr.12016 23586575

[49] Kondrup SV Anhoj KP Rodsgaard-Rosenbeck C Lund TB Nissen MH Sandoe P. Veterinarian's dilemma: a study of how Danish small animal practitioners handle financially limited clients. Vet Rec. (2016) 179:596. 10.1136/vr.103725 27811050

[50] AVMA. COVID-19: Veterinary Impacts and Responses. American Veterinary Medical Association. (2020). Available online at: www.avma.org (accessed September 6, 2021).

[51] Wild I Gedge A Burridge J Burford J. The impact of COVID-19 on the working equid community: responses from 1,530 individuals accessing NGO support in 14 low- and middle-income countries. Animals. (2021) 11:1363. 10.3390/ani11051363 34064832 PMC8151231

[52] Mcrobert CJ Hill JC Smale T Hay EM Van Der Windt DA. A multi-modal recruitment strategy using social media and internet-mediated methods to recruit a multidisciplinary, international sample of clinicians to an online research study. PLoS ONE. (2018) 13:e0200184. 10.1371/journal.pone.0200184 29979769 PMC6034855

[53] Byrnes C Ganapathy VA Lam M Mogensen L Hu W. Medical student perceptions of curricular influences on their wellbeing: a qualitative study. BMC Med Educ. (2020) 20:288. 10.1186/s12909-020-02203-4 32867759 PMC7457773

[54] Van Oosterhout C Hall N Ly H Tyler KM. COVID-19 evolution during the pandemic – implications of new SARS-CoV-2 variants on disease control and public health policies. Virulence. (2021) 12:507–8. 10.1080/21505594.2021.1877066 33494661 PMC7849743

