# Supplementary material for: Risk Factors Associated With Increased Ethically Challenging Situations Encountered by Veterinary Team Members During the COVID-19 Pandemic
Source: Front Vet Sci. 2021 Oct 25;8:752388. doi: 10.3389/fvets.2021.752388 (PMC8573112; doi:10.3389/fvets.2021.752388)
Supplement: Supplementary file 1 [file Table_1.DOCX]

Supplementary Table 1. Key to variable transformation to facilitate statistical analysis.

| **Category** | **Original variable** | **Transformed variable** |
| --- | --- | --- |
| Role | Veterinarian | Veterinarian |
|  | Animal health technician | Not veterinarian |
|  | Veterinary nurse | Not veterinarian |
|  | Other animal health professional | Not veterinarian |
| Gender | Female | Female |
|  | Male | Male |
|  | Other | Excluded from analysis due to small sample size. |
| Country | Australia | Australia and New Zealand |
|  | New Zealand | Australia and New Zealand |
|  | USA | USA and Canada |
|  | Canada | USA and Canada |
|  | Austria | Other |
|  | Belarus | Other |
|  | Cambodia | Other |
|  | China | Other |
|  | Denmark | Other |
|  | France | Other |
|  | Hong Kong | Other |
|  | Jamaica | Other |
|  | Lithuania | Other |
|  | Mexico | Other |
|  | Netherlands | Other |
|  | Republic of Ireland | Other |
|  | Singapore | Other |
|  | Spain | Other |
|  | Thailand | Other |
|  | United Kingdom | Other |
|  | Zimbabwe | Other |
| Hours worked | 0-10 | 0-30 |
|  | 11-20 | 0-30 |
|  | 21-30 | 0-30 |
|  | 31-40 | 31-40 |
|  | 41-50 | 41->50 |
|  | 50+ | 41->50 |
| Caseload | Companion animal practice clinical | Companion animal clinical practice |
|  | Mixed animal practice clinical | Other clinical practice |
|  | Exotic/unusual animal practice clinical | Other clinical practice |
|  | Zoo and/or wildlife practice clinical | Other clinical practice |
|  | Equine practice clinical | Other clinical practice |
|  | Practice management | Other clinical practice |
|  | Academia/teaching | Non-clinical role |
|  | Scientific research/laboratory animals | Non-clinical role |
|  | Government | Non-clinical role |
|  | Non-government organisation | Non-clinical role |
|  | Industry (e.g. pharmaceutical companies, food companies) | Non-clinical role |
|  | No longer a veterinarian | Non-clinical role |
|  | Other | Non-clinical role |
| Post-qualification ethics training | Continuing professional development | Yes |
|  | Sat on an institutional ethics committee | Yes |
|  | University coursework in an ethics or bioethics degree | Yes |
|  | Another form of ethics training | Yes |
|  | None | No |
| Confidence in resolving ethically challenging situations | Not confident at all | Not confident at all/underconfident |
|  | Underconfident | Not confident at all/underconfident |
|  | Confident enough that I can get by | Confident enough that I can get by |
|  | Reasonably confident | Reasonably confident/couldn’t be more confident |
|  | Couldn’t be more confident | Reasonably confident/couldn’t be more confident |
| Autonomy | Never | Never/rarely |
|  | Rarely | Never/rarely |
|  | Sometimes | Sometimes |
|  | Most of the time | Most of the time/always |
|  | Always | Most of the time/always |
